# Searching for bacterial plastitrophs in modified Winogradsky columns

**DOI:** 10.3389/frmbi.2024.1303112

**Published:** 2024-03-20

**Authors:** Fatai A. Olabemiwo, Claudia Kunney, Rachel Hsu, Chloe De Palo, Thaddeus Bashaw, Kendall Kraut, Savannah Ryan, Yuting Huang, Will Wallentine, Siddhant Kalra, Valerie Nazzaro, Frederick M. Cohan

**Affiliations:** ^1^ Department of Biology, Wesleyan University, Middletown, CT, United States; ^2^ Quantitative Analysis Center (QAC), Wesleyan University, Middletown, CT, United States

**Keywords:** plastic pollution, polyethylene strips, plastitrophs, Winogradsky columns, plastic bioremediation

## Abstract

**Introduction:**

Plastic pollution has surged due to increased human consumption and disposal of plastic products. Microbial communities capable of utilizing plastic as a carbon source may play a crucial role in degrading and consuming environmental plastic. In this study, we investigated the potential of a modified Winogradsky column (WC) to enrich Connecticut landfill soil for plastic-degrading bacteria and genes.

**Methods:**

By filling WCs with landfill soil and inorganic Bushnell Haas medium, and incorporating polyethylene (PE) strips at different soil layers, we aimed to identify bacterial taxa capable of degrading PE. We employed high-throughput 16S rRNA sequencing to identify the microbes cultivated on the plastic strips and the intervening landfill soil. We used PICRUSt2 to estimate the functional attributes of each community from 16S rRNA sequences.

**Results and discussion:**

After 12 months of incubation, distinct colors were observed along the WC layers, indicating successful cultivation. Sequencing revealed significant differences in bacterial communities between the plastic strips and the intervening landfill-soil habitats, including increased abundance of the phyla Verrucomicrobiota and Pseudomonadota (néé Proteobacteria) on the strips. Based on inferred genomic content, the most highly abundant proteins in PE strip communities tended to be associated with plastic degradation pathways. Phylogenetic analysis of 16S rRNA sequences showed novel unclassified phyla and genera enriched on the plastic strips. Our findings suggest PE-supplemented Winogradsky columns can enrich for plastic-degrading microbes, offering insights into bioremediation strategies.

## Graphical Abstract

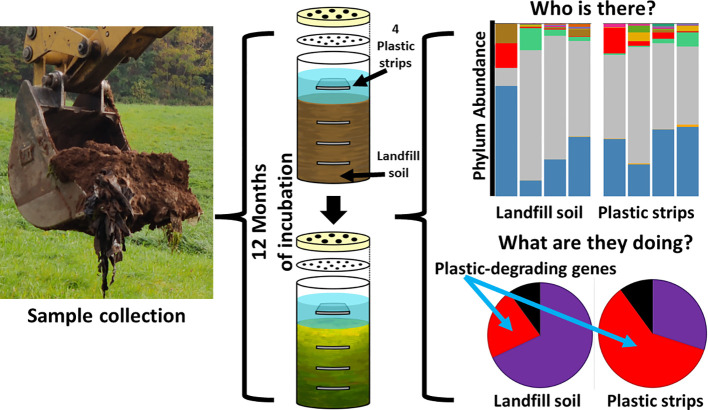

## Highlights

• Modified Winogradsky columns were used to enrich landfill soil for plastic-degrading microbes by incorporating polyethylene strips.
• Polyethylene strips increased the abundance of the phyla Verrucomicrobiota and Pseudomonadota compared to intervening landfill soil in the columns.
• Proteins predicted to be most abundant in polyethylene strip communities were implicated in plastic degradation.
• Phylogenetic analysis revealed novel candidate phyla and genera enriched on PE strips.

## 1 Introduction

Plastic pollution has reached unprecedented levels as human consumption and disposal of plastic products have skyrocketed (Piehl et al., 2018). Plastic pollution has received increased attention in recent years as a significant environmental concern because plastic can pollute air, soil, and water, and then harm living organisms (Smith et al., 2018). Once the plastic is released, it can remain in the environment for decades or longer, and break down into small pieces known as microplastics and nanoplastics (Zhang et al., 2021). The indefinitely long presence of degraded plastic debris threatens food security (Rillig et al., 2019; Zhang et al., 2020) because humans can unknowingly ingest nanoplastic particles through foods such as fish (Thiel et al., 2018), mussels (Wright and Kelly, 2017), vegetables, and other crops (Conti et al., 2020). Plastic debris also threatens food security by harming plants and soil microorganisms in agricultural environments, leading to reduced crop yields and declines in soil fertility (FAO and WHO, 2021). Therefore, there is an urgent need to develop strategies to reduce plastic pollution and its consequences.

Environmental scientists have attempted to combat plastic pollution through biodegradation, a process employing bacteria to degrade plastic into monomeric subunits (Shah et al., 2008; Ghosh et al., 2013). Many studies have isolated bacteria and other microbes, such as plastic-colonizing fungi, from the environment, and have used them for biodegradation studies (Hadad et al., 2005; Yoshida et al., 2016; Dang et al., 2018; Dey et al., 2020). The term plastisphere, analogous to the rhizosphere, has been used to describe the diverse microbial communities colonizing environmental plastic (Amaral-Zettler et al., 2020). Plastispheres have been analyzed from soil and water environments (Oberbeckmann et al., 2018; Ogonowski et al., 2018; Rüthi et al., 2020). Terrestrial and aquatic plastispheres appear to sustain a diversity of plastic-degrading bacteria, but there is limited data from terrestrial environments. Expanding our knowledge of plastic-degrading bacterial diversity could advance bioremediation efforts to manage plastic waste.

Polyethylene (PE) was the focus of this study due to its global dominance as the most abundantly produced recyclable plastic (Conk et al., 2022), and because it accounts for a high proportion of plastic waste polluting the environment (Wyss et al., 2023). PE is a sturdy synthetic plastic waste that does not break down completely in the environment (Conk et al., 2022), yielding microplastic and nanoplastic debris that persists in the environment for extended periods (Dey et al., 2020). Here, we explore the bacteria of terrestrial plastispheres by employing a modified Winogradsky column. A Winogradsky column is a device used to enrich soil microorganisms of interest, including novel bacteria that can degrade certain compounds, including plastics (de Sousa et al., 2012; Cantillo-González et al., 2022). These columns are named after Russian microbiologist Sergei Winogradsky, who developed the technique in the late 19th century (Winogradsky, 1890; Schlegel and Bowien, 1989). Winogradsky columns can function as models for microbial ecosystems where the conditions can be controlled, in contrast to microbial communities at natural sampling sites (Esteban et al., 2015; Babcsányi et al., 2017). In a Winogradsky column, the different niches and microbial communities formed as a result of chemical and oxygen gradients can be visualized and stratified; the layers that would naturally exist within a few millimeters are expanded to decimeters (Rogan et al., 2005; Rundell et al., 2014). The topmost layer contains high oxygen levels, leading to the cultivation of aerobic bacteria. Oxygen levels decrease with greater depth, allowing enrichment of microaerophilic and anaerobic bacteria.

Winogradsky columns have previously been used to isolate potential plastic-degrading bacteria. Our study builds on earlier efforts by inoculating our Winogradsky columns with landfill soil that had been exposed to plastics for decades. Also, whereas previous efforts explored only the top and middle layers of a Winogradsky column (Kotova et al., 2021), we placed polyethylene (PE) strips horizontally at four depths in a column. This design allowed us to compare the bacterial communities growing on the plastic (PE) strips versus the intervening landfill soil between strips, across different depths. After extended culture in Winogradsky columns, we collected samples from the PE strips and the landfill soil.

We characterized the diversity of bacteria from each environment type by high-throughput sequencing of the 16S rRNA gene. We aimed to develop and validate our novel method for its ability to yield plastic-degrading bacteria. Our approach was first to determine whether the plastic strips would yield a different bacterial community from the adjacent landfill soil in the columns. We then determined whether the plastic strips would yield a greater level of plastic-degrading function than the adjacent soil, using the PICRUSt2 algorithm (Douglas et al., 2020) to infer genome content from 16S rRNA sequences. We propose the term “plastitroph” for bacteria with the ability to be nourished through plastic degradation, a term we believe better characterizes bacterial consumption of plastic than “plastivore,” which implies animal-like devouring of plastic.

## 2 Materials and methods

### 2.1 Sampling sites and soil collection

With permission and assistance from the Town of Portland, Connecticut, we collected a soil sample from the decommissioned and capped landfill at the Portland Transfer Station and Recycling Center on October 29, 2021. Collection was at a four-foot depth from a location (41°34’18″N, 72°36’39”W) where plastic and other waste had been buried for over 40 years, according to the caretaker. The collected soil samples were placed in a sterile 25 L plastic container covered with aluminum foil. The samples were then transported to the Biology Department at Wesleyan University.

### 2.2 Preparation of modified Winogradsky columns

**Figure 1:**
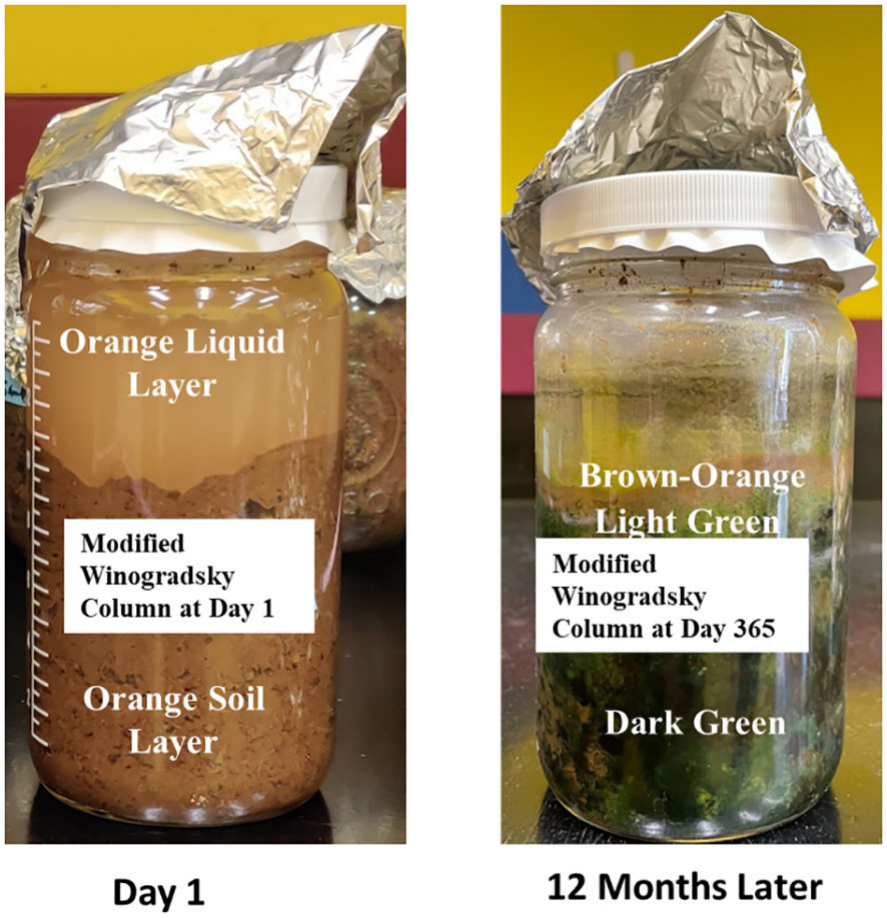
Modified Winogradsky columns enriched with plastic strips at different layers.

Four replicate Winogradsky columns were prepared aseptically in a laminar flow hood to avoid contamination. The Winogradsky design was modified so that, beyond the carbon resources in the soil, polyethylene was provided as supplemental carbon, as strips at different layers (**Figure 1**). First, a PE sheet was cut into strips of size 3 x 2 cm, which were individually weighed. The PE strips were placed in 70% ethanol, rinsed in sterile water thrice, and aseptically air-dried in a laminar flow hood. Second, we aseptically scooped soil into a 500 mL graduated glass bottle (the Winogradsky column) up to the 100 mL mark and placed a sterile PE strip horizontally on the soil. Adding more soil up to the 200 and 300 mL marks, we added additional strips at the 200 and 300 mL marks, and then finally, we added more soil to the 350 ml mark. Sterile Bushnell Haas (BH, Difco) broth was added to the column to wet the soil throughout the column and to form an aqueous layer up to 100 ml above the settled soil. We added a fourth PE strip to the top liquid layer.

BH Broth is an inorganic medium used to enrich for bacteria that can utilize hydrocarbons (Khandare et al., 2021). BH broth contains all the required nutrients (MgSO_4_, CaCl_2_, KH_2_PO_4_, K_2_HPO_4_, FeCl_3_, and NH_4_NO_3_) for the growth of many bacteria, except carbon. The PE strips served as an additional source of carbon beyond the landfill soil.

Finally, we sealed the columns with a 0.2 µm nylon membrane filter to prevent microbes from entering the column, while allowing for airflow. We covered each column with a bottle cap that was drilled with 6 tiny holes (2 mm) by the IDEA lab, Wesleyan University.

Each column was incubated at room temperature for 12 months.

### 2.3 Sample collection from Winogradsky columns

After 12 months of incubation, samples were aseptically collected from the landfill soil above and below each PE strip and from the biofilm growing on each strip. The strip from the top liquid layer was picked directly using sterile tweezers. The landfill soil was gently scooped to expose the soil at the middle of each soil layer above and below each strip. The soil was wet enough to be sampled with a pipette, and 500 µL was collected from the midpoint of each layer. For the strips buried in the soil, we gently scooped out the soil with a sterile spatula until the strip was located. The strip was then removed using a sterile tweezer. This stage was repeated for the second and third soil layers. Each strip was placed in a sterile test tube containing 5 ml of sterile BH broth and was vortexed for 20 min to remove the cells and small soil particles attached to the plastic surface. The plastic strips were then discarded. 500 µL of each sample plus 500 µL of sterile glycerol were placed in Eppendorf tubes, and were frozen at -80°C until DNA extraction.

### 2.4 DNA extraction, 16S rRNA amplification, and sequencing

From each sample, 5 ml of suspension was processed for genomic DNA extraction. According to the manufacturer’s instructions, the DNA was isolated using ZymoBIOMICS DNA isolation kits (Zymo Research Corporation, Irvine, CA, USA). The V3-V4 region of the 16S rRNA gene was amplified using universal bacterial-specific primers 341F(5’- CCT ACG GGN GGC WGC AG-3’) and 785R (5’- GAC TAC HVG GGT ATC TAA TCC-3’) (Parada et al., 2016; Fadeev et al., 2021) via polymerase chain reaction (PCR). Each reaction contained 1 μL genomic DNA, 10 μL PCR master mix at a 2X final concentration, 0.5 μL of each primer (forward and reverse) at a final concentration of 10 μM, and 13 μL sterile molecular-grade water. The PCR cycling conditions were as follows: 94°C for 3 min to denature the DNA, followed by 35 cycles at 94°C for 45 s, 50°C for 60 s, and 72°C for 90 s, with a final extension at 72°C for 10 min to ensure complete amplification. PCR was performed in triplicate on each sample, then combined and cleaned using a MoBio UltraClean PCR Clean-up Kit (Costello et al., 2009).

Sequencing was performed on an Illumina MiSeq platform at 2 × 250 bp to generate paired-end reads at Quintara Bioscience (Cambridge, MA, USA). Raw reads were deposited in the National Center for Biotechnology Information (NCBI) database under project number PRJNA1014774.

### 2.5 Taxonomic classification of 16S rRNA amplicons

All bioinformatic analyses were conducted on the Nephele microbiome platform (Weber et al., 2017) using a customized pipeline based on the DADA2 workflow (version 1.16) (Callahan et al., 2016). Cutadapt (version 4.1) trimmed the primers from the raw reads. We used only single-end (forward) reads for these analyses because there were insufficient reads to create a minimum overlap, following Callahan et al. (2021); Ramakodi (2021); Ramakodi (2022); Koike et al. (2023). We then filtered and trimmed reads with low-quality bases and removed reads with less than 75 bp lengths. To complete the preprocessing of raw reads, the DADA2 software identified chimeric reads and removed them.

We dereplicated identical sequences, creating a library of unique amplicon sequence variants (ASVs). We classified the ASVs to taxon using the SILVA 16S rRNA gene database (version 138.1) and the Ribosomal Database Project (RDP; version 18). Our analysis focused on ASVs assigned to either bacteria or archaea; ASVs assigned to eukaryotes, mitochondria, or chloroplasts were removed from the dataset. For quality control against sequencing errors, we included only ASVs with counts of five or more in our analyses.

### 2.6 Taxonomic comparisons of Winogradsky communities from PE strips versus intervening landfill soil

Unless otherwise indicated, all statistical analyses and data visualizations were performed using the R statistical package (version 4.2.2) (RCoreTeam, 2020). In this study, statistical tests were considered significant if the P-value was less than 0.05 unless otherwise specified. If necessary, P-values were corrected for multiple testing using the Benjamini-Hochberg false discovery rate method (Benjamini and Hochberg, 1995). ASV counts for each sample were managed using phyloseq (version 1.40.0), and plots were generated with ggplot2 (version 3.4.2).

**Figure 2:**
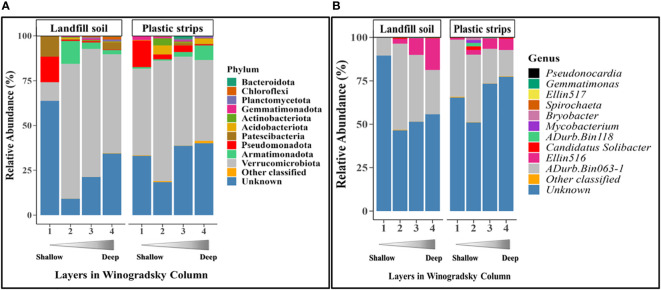
Relative abundance of the top ten most-abundant classified bacterial **(A)** phyla and **(B)** genera, as well as other classified and unknown taxa, for each layer of plastic strips and landfill soil.

For each depth separately, we compared the pool of all plastic strips to the pool of all landfill soil samples (pooled over all four columns). Here, we compared the relative abundance of the ten most abundant taxa (at the phylum and genus ranks) and tested for significance using the Chi-square contingency test (**Figure 2**).

We quantified the α-diversity of ASVs by the Chao1 and Shannon indices for each of the 32 communities as measures of richness and evenness. These include 4 replicate columns X 2 substrate types (plastic strips versus landfill soil) X 4 layers. We then calculated the effect sizes for both Chao1 and Shannon indices using the Cohen’s d effect size (Equation 1) to determine the magnitude of the variations between plastic strips and landfill soil communities.


(1) $$d=\frac{M_{ps}-M_{ls}}{Pooled\;standard\;deviation}$$

Where M_ps_ represents the mean Chao1 or Shannon indices for plastic strips; Mls represents the mean Chao1 or Shannon indices for landfill soil. Pooled standard deviation was calculated following Equation 2:


(2) $$Pooled\;standard\;deviation=\sqrt{\frac{SD_{ps}{\;}^{2}+SD_{ls}{\;}^{2}}{2}}$$

Where SD_ps_^2^ is the standard deviation of Chao1or Shannon for plastic strips; SD_ls_^2^ is standard deviation of Chao1 or Shannon for landfill soil.

We next identified individual ASVs that were significantly more abundant on the pool of all plastic strips versus landfill soil samples. For a given ASV, we compared the mean relative abundance on plastic strips versus the mean relative abundance on landfill soil, using the differential abundance function of Limma-Voom with trimmed mean of M-values (TMM) normalized using the Limma package in R (Baik et al., 2020) (**Supplementary Figure S1**). The TMM normalization method was used to address sequencing depth variations between samples, ensuring the abundance levels were normalized based on the distribution of ASV counts. First, we normalized the ASVs using the TMM method, which corrects for variations in sequencing depth between samples by normalizing the abundance levels based on the distribution of their ASV counts. Second, we fitted linear models with Limma and Voom to the normalized data and then performed a test to identify ASVs with significantly greater abundance on plastic strips relative to landfill soil based on log2-fold changes using edgeR. We considered ASVs and taxa significantly different between plastic and landfill soil samples only if the false discovery rate (adjusted p-value) was < 0.05.

### 2.7 Predicting the biochemical functions of plastic communities using PICRUSt2

**Figure 3:**
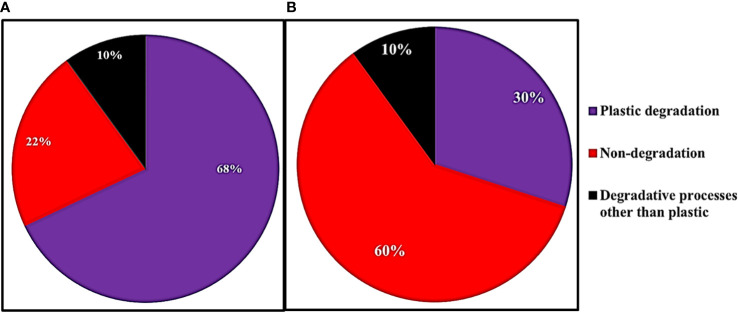
The fraction of genes associated with plastic degradation, other degradation, and non-degradation for the 50 genes with the **(A)** highest and **(B)** lowest ratios of relative abundance on plastic strips versus landfill soil.

We used the PICRUSt2software (Douglas et al., 2020) to predict the biochemical functions and pathways coded by the genome of each ASV, based on the functions typically held by the ASV’s taxon. We aimed to quantify the differences in biochemical function between the Winogradsky communities on plastic strips versus landfill soil. The relative abundance of a given gene in a given community sample (in one replicate column) was quantified by pooling the abundance of all ASVs that were predicted to have the gene, divided by the total number of all ASV reads. We averaged the relative abundance of each gene for a given community type (eight types of communities: the four layers times the two types of surfaces, plastic strips versus landfill soil) over all four column replicates (**Figure 3**). We rank-ordered the genes by the ratio of their average relative abundance in plastic strip communities versus that in landfill soil. We log-transformed the ratio of these average relative abundances.

**Figure 4:**
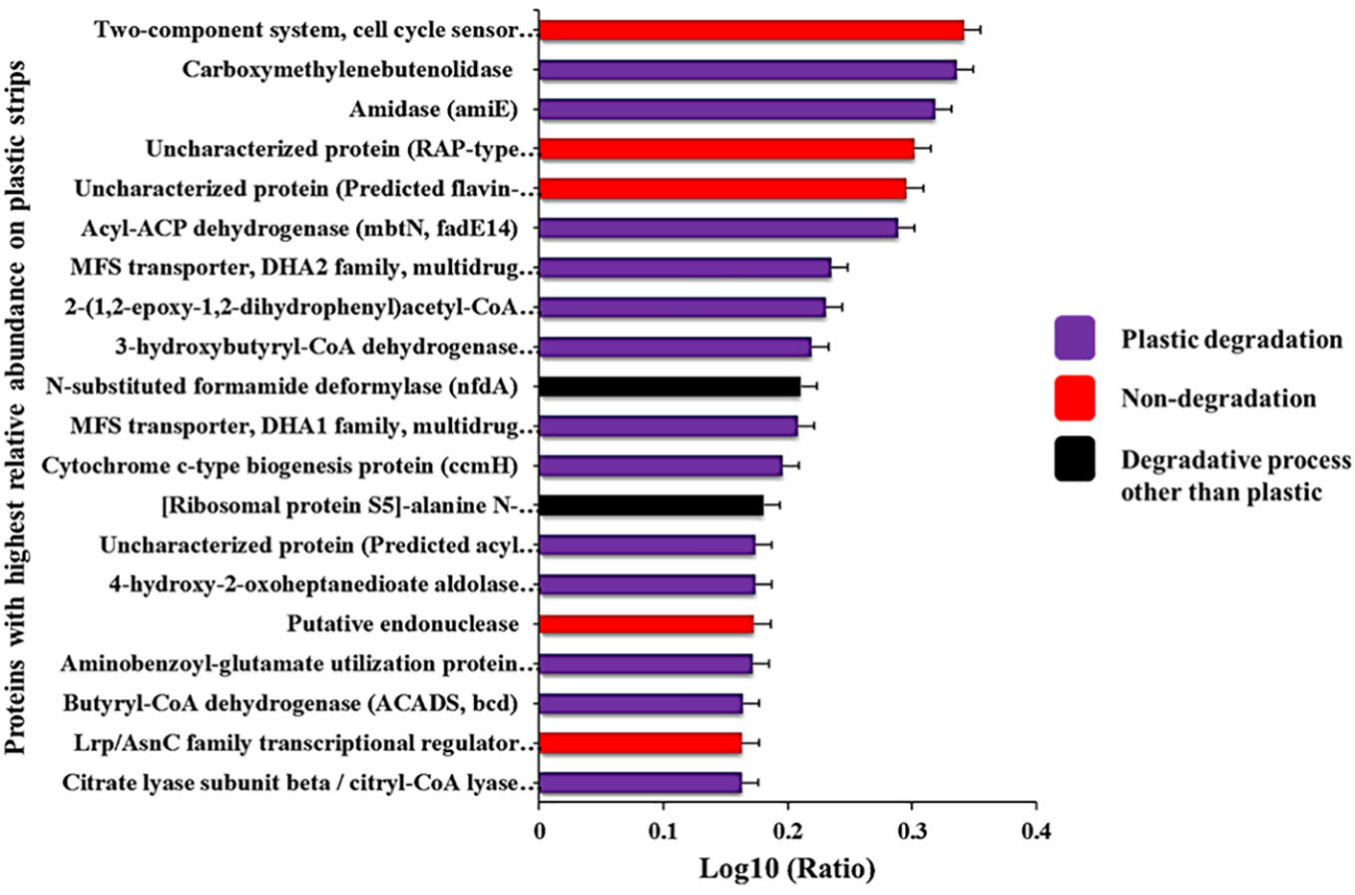
Proteins with the highest ratios of abundance on plastic strips versus landfill soil. The full names of these proteins are given in **Supplementary Table S1**.

We then aimed to characterize the functions of the genes with the greatest and least relative abundance on plastic strips versus landfill soil (**Figure 4**). For the top 50 genes in each category, we searched the Kegg database for the various pathways each gene belonged to. Each gene was then classified into plastic degradation (if at least one of its pathways involved plastic degradation), degradative processes for molecules other than plastics, or non-degradation.

### 2.8 Phylogenetic analysis

**Figure 5:**
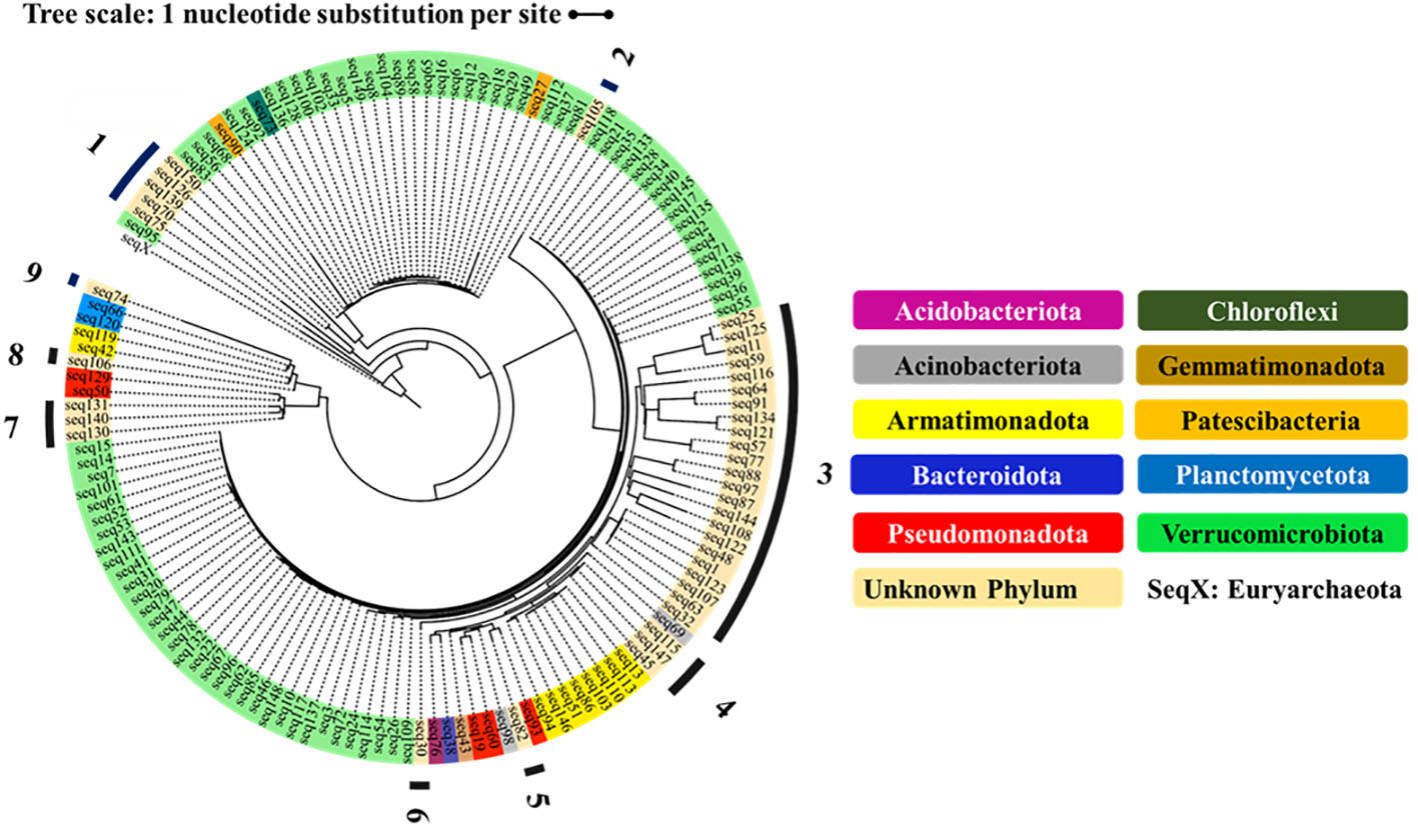
Phylogenetic relationship of 150 ASVs, classified to phyla and (rooted to the Euryarchaeota of the Archaea). The outgroup Seq X represents the Euryarchaeota of the Domain Archaea. The unknown phyla are numbered 1-9.

To compare bacterial communities of plastic strips and landfill soil, we constructed phylogenetic trees of the 150 most abundant ASVs, rooting trees with an archaeal outgroup (*Halobacterium salinarum strain* 91-R6) (**Figure 5**). First, we identified the closest relatives of each ASV from public databases and aligned ASVs to relatives using MUSCLE v0.0.11 (Edgar, 2004; Cuccuru et al., 2014). After manually reviewing alignments, we estimated a maximum likelihood phylogeny with RAxML v8.2.12 (Stamatakis, 2014), selecting the best substitution model for each dataset. We assessed tree robustness via ultrafast bootstrapping (2,000 replicates) and Bayesian-like transformation of the approximate likelihood ratio test in RAxML. Second, we generated an unrooted tree containing 8 ASVs unclassified to domain (of the top 150) with reference sequences from Archaea (*H. salinarum* 91-R6), Bacteria (*Collimonas fungivorans* Ter6), and Eukaryota (*Basidiobolus heterosporus* CBS 311.66) (**Supplementary Figure S2**). The alignments used in this analysis are available on request. Third, we created a rooted tree (*Chloroflexus aurantiacus* outgroup) of unclassified ASVs at the genus-level for each of the top 3 phyla (Armatimonadota, Pseudomonadota, Verrucomicrobia) (**Supplementary Figures S3**–**S5**). For Armatimonadota, 16S rRNA sequences of known genera were included from NCBI (*Capsulimonas corticalis* AX-7, *Fimbriimonas ginsengisoli* Gsoil 348, *Chthonomonas colidirosea* T49, *Armatimonas rosea* YO-36) (**Supplementary Figure S3**). For trees representing the Pseudomonadota and Verrucomicrobia phyla, we included both classified and unclassified ASVs (**Supplementary Figures S4** and **S5**, respectively). Specifically, we incorporated ASVs that were unclassified at the genus level as well as those that were unclassified at the genus level as well as those that were classified to known genera. This comprehensively captured phylogenetic diversity within these phyla for our analysis. All trees were visualized on iTOL (Letunic and Bork, 2021). Alignments used are available upon request.

**Figure 6:**
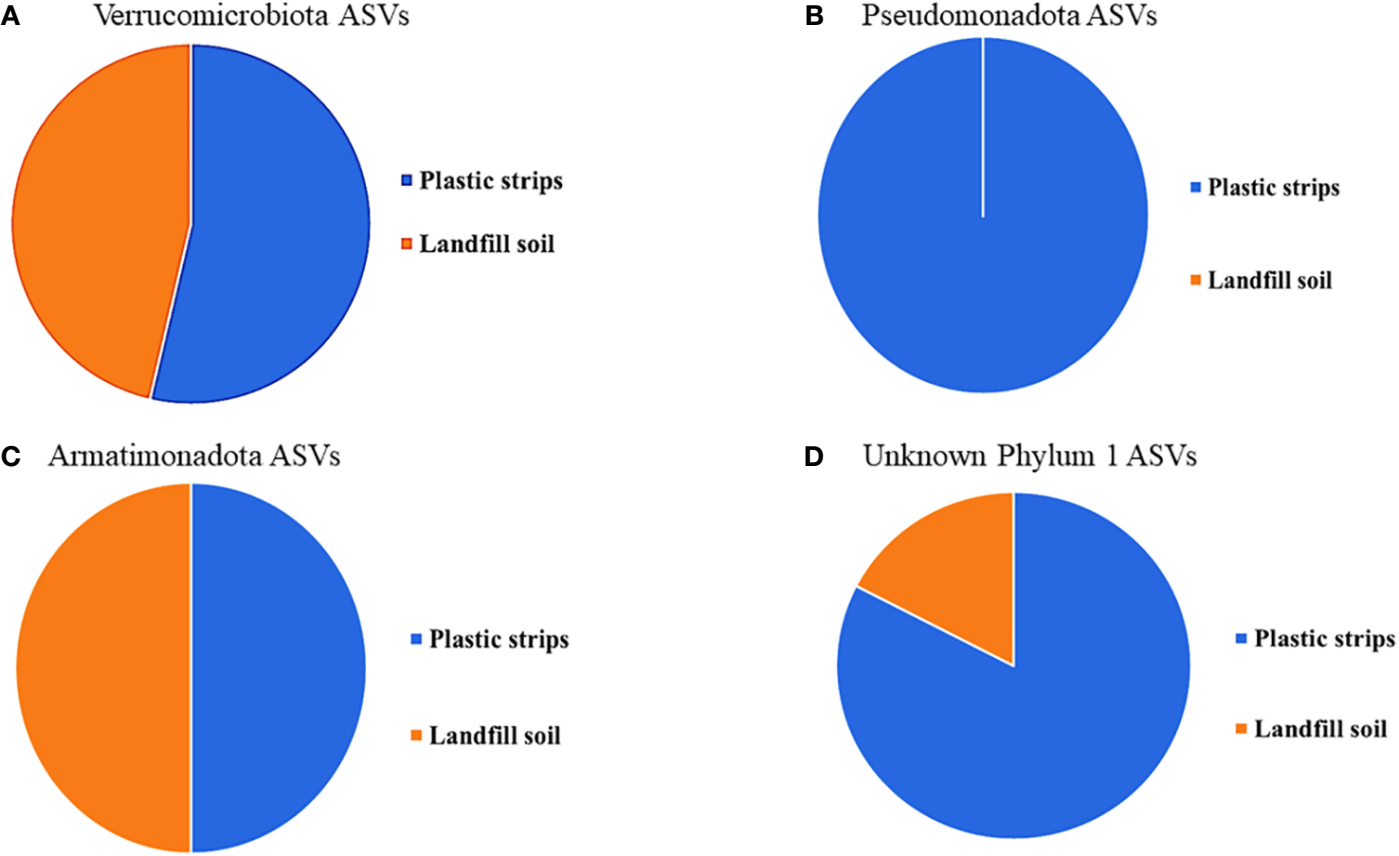
For each phylum with at least five ASVs, **(A)** Verrucomicrobiota, **(B)** Pseudomonadota, **(C)** Armatimonadota, and **(D)** Unknown Phylum 1, the fraction of organisms from plastic strips versus landfill soil.

For each phylum with at least five ASVs, we showed the fraction of organisms from plastic strips versus landfill soil (**Figure 6**).

## 3 Results

### 3.1 Visual examination of the Winogradsky columns

At the start of incubation on day 1, we observed uniform layering across the Winogradsky columns, with orange liquid over a homogeneous, orange soil layer. After 12 months of incubation, we observed dramatic color changes ranging from brown-orange to light and dark green along the range of depths on the inside walls of the Winogradsky columns. The colors confirmed that our approach successfully cultivated bacteria from the landfill soil at all depth levels and oxygen concentrations. Through nurturing complex microbial niches, our long-term column system mirrored previous findings that such setups foster pronounced differentiation of communities and geochemical zones along oxygen and chemical gradients (Esteban et al., 2015).

### 3.2 Quality control and filtering of sequence reads

We processed a total of 342,987 single-end Illumina raw reads obtained from 32 samples (8 community types times four replicate columns). After preprocessing and a quality filtering stage, we obtained 310,992 valid reads and then removed low-quality reads and chimeras to generate a total of 225,675 high-quality reads. From this set, we identified 2,463 ASVs over all samples. Finally, we filtered out all sequence variants with counts < 5 to obtain 2,066 ASVs used in further analyses.

### 3.3 Community compositions on plastic strips versus landfill soil

We compared the most abundant taxa at the phylum and genus level in plastic strips versus landfill soil samples (**Figure 2**). At the phylum level, 10 taxa were present in high abundance over the pool of both types of samples: Verrucomicrobiota (53.6% averaged over both samples), Pseudomonadota (4.12%), Patescibacteria (1.92%), Bacteriodota (0.60%), Armatimonadota (2.30%), Actinobacteriota (0.91%), Chloroflexi (0.20%), Gemmatimonadota (0.75%), Planctomycetota (0.40%), and Acidobacteriota (1.15%). Low-abundance phyla included the Bacillota, Desulfobacterota, Fibrobacterota, Nitrospirota, Spirochaetota, Elusimicrobiota, Sumerlaeota, FCPU426, WS4, and Myxococcota (based on a pool of both types of samples:< 0.1%). At the genus level, the two most abundant genera over the pool of both plastic strips and landfill soil samples were the candidate genera (*Adurb.Bin063-1*: 31.6% and *Ellin 516:* 27.8%).

The communities from plastic strips differed from landfill soil at each depth, as shown by the relative abundances of the ten most highly abundant phyla (**Figure 2A**). Here, we present the most prominent and interesting differences between the plastic-strip and landfill-soil communities. For each layer tested separately, the phylum distributions were significantly different for the pool of plastic strips versus the pool of landfill soil samples (χ^2^ = 3320.5, p< 2.2e-16; χ^2^ = 5704.5, p< 2.2e-16; χ^2^ = 4073.1, p< 2.2e-16; χ^2^ = 5121.4, p< 2.2e-16, for Layers 1-4 respectively) For example, in the top layer (Layer 1), the phylum Verrucomicrobiota was more abundant in the plastic strip than landfill soil samples (56.7% in plastic strips versus 17.1% in landfill soil). In Layer 2, Actinobacteriota (1.89%) was present only in the plastic strips, and Acidobacteriota was much more abundant in plastic strips (4.33%) than in landfill soil (0.14%). The phyla Pseudomonadota (1.83%) and Patescibacteria (1.30%) were present only in plastic strips in Layer 3. For Layer 4, phylum Armatimonadota was more abundant in plastic strips (8.27%) than landfill soil (1.05%), and Acidobacteriota and Gemmatimonadota were present only on plastic strips.

At the genus level, we observed four genera (*Adurb.Bin118, Mycobacterium, Candidatus* Solibacter*, and Bryobacter*) that were more abundant in the plastic strips than in the landfill soil samples of Layer 2 (**Figure 2B**).

### 3.4 Differences in diversity measures in plastic versus landfill soil samples

We estimated the alpha diversity at the ASV level for each of the 32 communities using the Chao1 and Shannon indices. Averaged over 16 communities each, we found that the diversity levels of richness and evenness in plastic strips versus landfill soil were similar, for both Chao1 (plastic: 109.3 vs. landfill soil: 88.4) and Shannon (plastic: 3.24 vs landfill soil: 2.98). The Cohen’s d effect size provided more insight into the magnitude of the differences observed. For Chao1, d = 0.22 implies that plastic strip bacterial communities tend to have moderately higher (~22%) observed richness than landfill soil. Similarly, the Shannon’s d = 0.29 indicates that plastic strip bacterial communities tend to have moderately greater (~29%) diversity than those inhabiting adjacent landfill soil.

We tested whether the relative abundance of each ASV was different on plastic strips versus landfill soil (**Supplementary Figure S1**). Seven ASVs showed a significantly different relative abundance on plastic strips versus landfill soil: five ASVs were significantly more abundant in plastic-strip samples, and two were significantly more abundant in landfill-soil samples.

### 3.5 Functional diversity of bacterial communities on plastic-strip and landfill-soil samples

We used PICRUSt2 to predict (from 16S rRNA abundances) the average relative abundance of genes in plastic-strip and landfill-soil samples. For each gene, we quantified the ratio of relative abundance in plastic strips to landfill soil. For the genes with the top 50 and bottom 50 ratios, we searched the KEGG Orthology database for the KEGG pathways associated with these genes. Here, we classified the genes/proteins into plastic degradation, degradative processes other than plastics, and nondegradation. Here, we observed that among the top 50 genes most highly associated with plastic strips, 68%, 10%, and 22% were associated with plastic degradation, degradative processes other than plastics, and nondegradation, respectively (**Figure 3A**). In contrast, among the 50 genes least associated with the plastic strips, we observed that 30%, 60%, and 10% were associated with plastic degradation, degradative processes other than plastics, and nondegradation, respectively (**Figure 3B**) (χ^2^ = 16.2, df=2, *p=0.0003*). We also report the top 20 genes with the highest ratios of abundance on plastic strips versus landfill soil (**Figure 4**). 65% of these genes were involved in plastic degradation.

### 3.6 Phylogenetic tree shows novel phyla

The phylogenetic tree in **Figure 5** shows the diversity of the top 150 most abundant ASVs in our study. The tree shows nine ASV clusters that were not classified to a known phylum; these each potentially represent a novel phylum. The four phyla with five or more ASVs (Verrucomicrobiota, Armatimonadota, Pseudomonadota, and ‘Unknown Phylum 1’) were each more abundant on the plastic strips than on the landfill soil (**Figure 6**). This compares to a 50.0% average relative abundance on plastic strips over all ASVs.

## 4 Discussion

Our study addresses the accumulation of polyethylene in soils, a problem exacerbated by the increasing use and disposal of polyethylene (PE), its recalcitrance to biodegradation, and the limited diversity of bacteria that are known to degrade this plastic (Pathak and Navneet, 2017). To date, there are fewer than 20 bacterial genera known to degrade PE (Restrepo-Flórez et al., 2014; Pathak and Navneet, 2017). We have attempted a novel approach to vastly broaden the known diversity of bacteria that can degrade PE. Our approach began with sampling soil laden for decades with decaying plastic, followed by cultivating the soil in Winogradsky columns supplemented with PE sheets at different layers of oxygenation, and then surveying the diversity of bacteria so cultivated with high-throughput amplification and sequencing of DNA. Finally, we compared the plastic-strip and landfill-soil communities from the columns to test whether the plastic strips were enriching for plastic-degrading taxa and genes.

We aimed to test whether adding the PE sheets at different layers could enrich PE-degrading bacteria, even when starting with soil already laden with PE. We found that the plastic-strip versus landfill-soil communities within the Winogradsky columns were significantly different in composition at the phylum and genus levels in each layer. The plastic strips in the first layer had higher diversity than the landfill soil (Cohen d= 1.3). While both habitats became less diverse with depth, plastic communities consistently maintained higher richness. This pattern may reflect plastic-associated niches sustaining greater functional potential. For example, Verrucomicrobiota was over three times more abundant on the plastic strips than on landfill soil samples in the top layer, and in the second layer, the phylum Acidobacteriota was 30 times more abundant on the plastic strips. Previous studies have also found Verrucomicrobiota to be a dominant phylum in plastic-degrading communities (McCormick et al., 2014; Amaral-Zettler et al., 2020). Bacterial species from this phylum may possess unique capabilities to degrade polyethylene (De Tender et al., 2015). Several genera were also more abundant on the plastic strips in the second layer. These results demonstrated that adding new plastic to soil that was likely already enriched for PE degraders (at the landfill) could further alter the frequencies of bacterial taxa.

To determine whether the plastic strips increased the abundance of plastic-degrading genes, we utilized a predictive approach based on 16S rRNA sequencing rather than the more costly metagenomic sequencing (Lombard et al., 2014; Moon et al., 2018). Specifically, we used the bioinformatics tool PICRUSt2 to infer the genomic content and relative gene abundances within the bacterial communities growing on the plastic strips versus the landfill soil (Douglas et al., 2020). This powerful and economical approach allowed us to identify the key functional differences between the Winogradsky communities on PE strips and landfill soil.

PICRUSt2 estimates gene content by leveraging existing genomic databases to predict which genes are present in organisms identified via 16S rRNA taxonomy (Douglas et al., 2020). Using this approach, we calculated each gene’s predicted abundance, pooled across all detected taxa known to contain that gene. We then compared plastic strips versus soil samples by determining the ratio of relative abundance for each gene. When ranked by this ratio, the top 50 genes enriched on plastic strips were remarkably distinct from the bottom 50 enriched in the soil communities. Strikingly, we found that 68% of the top 50 plastic-associated genes were implicated in plastic degradation pathways, over twice the proportion (30%) for the bottom-50 ranked genes associated with soil. These results indicate that supplementing the Winogradsky columns with plastic strips selected for microbial taxa that contain more plastic-degrading genes in their genomes. The enhanced potential for plastic biodegradation may enable these microbes to metabolize the PE strips as a carbon and energy source (Restrepo-Flórez et al., 2014). In summary, despite starting with soil historically contaminated with plastic, we could still further enrich for plastic-degrading genes by adding PE strips in a Winogradsky column.

We recognize limitations inherent to PICRUSt2 that require consideration when interpreting our functional predictions. As PICRUSt2 relies on existing genomic databases, novel taxa lacking sequenced genomes will not contribute to predictions of functional potential (Douglas et al., 2020). Nevertheless, PICRUSt2 has been used successfully to infer abundance of genes related to polyethylene degradation (Wright et al., 2021). PICRUSt2 remains a valuable tool for comparing functional potential across communities when applied judiciously (Toole et al., 2021). In our future work, metagenomics will provide a more direct, albeit more costly, quantification of functional genes.

Finally, our data provide encouraging evidence that we have discovered novel bacterial lineages with potential PE-degrading capabilities. Our phylogenetic analysis indicated nine distinct clusters that may each represent a novel phylum, as they did not match any classified phylum. One unknown phylum was approximately 6-fold enriched on the plastic strips compared to the soil samples, suggesting possible plastic degradation capacity. Within the known phyla Verrucomicrobiota, Pseudomonadota, and Armatimonadota, we discovered numerous unclassified genera that were substantially more abundant on plastic strips than on soil. Specifically, 58 novel genera in Verrucomicrobiota, 46 novel genera in Pseudomonadota, and 37 novel genera in Armatimonadota were over two-fold enriched on plastic strips. These results significantly expand the known diversity of bacteria associated with PE degradation beyond the fewer than 20 genera previously reported (Pathak and Navneet, 2017).

Our findings highlight the power of culture-independent techniques like 16S profiling for discovering novel diversity with biotechnology applications (Parks et al., 2018). However, we suggest further studies to characterize the novel taxa discovered here and to confirm their roles in PE degradation. Targeted isolation and growth experiments with representative taxa under controlled conditions could elucidate degradation pathways and kinetics. Gene expression analysis may reveal induction dynamics for relevant enzymes and pathways (Viljakainen and Hug, 2021). Enzymatic assays could validate predicted functions from our proteomics data (Zhang et al., 2023). Isotopic labeling coupled with metabolomics could track substrate fate through isolates and complex communities. Such targeted investigations combining multi-omic analyses with isotopic and enzymatic techniques would complement this initial characterization and validate potential PE-degrading capabilities.

## Data availability statement

The datasets presented in this study can be found in online repositories. The names of the repository/repositories and accession number(s) can be found below: https://www.ncbi.nlm.nih.gov/, PRJNA1014774.

## Author contributions

FO: Funding acquisition, Conceptualization, Formal analysis, Investigation, Project administration, Supervision, Writing – original draft, Writing – review & editing, Data curation, Methodology, Validation, Visualization. CK: Investigation, Writing – review & editing. RH: Investigation, Writing – review & editing. CD: Investigation, Writing – review & editing. TB: Investigation, Writing – review & editing. KK: Investigation, Writing – review & editing. SR: Investigation, Writing – review & editing. YH: Investigation, Writing – review & editing, Writing – original draft. WW: Investigation, Writing – review & editing, Formal analysis. SK: Formal analysis, Investigation, Writing – review & editing, Visualization. VN: Formal analysis, Investigation, Visualization, Writing – review & editing. FC: Formal analysis, Investigation, Visualization, Writing – review & editing, Conceptualization, Funding acquisition, Project administration, Resources, Supervision, Writing – original draft.

## Supplementary material

Supplementary Figure 1 Individual ASVs that are significantly different in their relative abundances in plastic strips versus landfill soil. Five ASVs **(A, B, E-G)** showed significantly greater abundance in plastic-strip samples, and two **(C, D)** were significantly more abundant in landfill-soil samples. Relative abundance refers to the proportion of sequencing reads assigned to either plastic strips or landfill soil for each ASV, averaged over all plastic-strip (16) or landfill-soil (16) communities sampled in the columns. Differences in relative abundance were tested by the Wilcoxon Rank test and FDR adjusted p-value< 0.05. The substantially greater representation of five of the ASVs on plastic strips suggests they may have plastic-degrading capabilities. The ASVs indicated here with greater relative abundance on plastic strips represent novel genera within the Verrucomicrobiota **(A, B)** and the Pseudomonadota **(G)**, or a novel phylum **(E, F)** Taxonomic classification and differential abundance analysis expand the known diversity of microbes associated with polyethylene degradation. Asterisks (*) represents the outliers for each ASV. The community type with greater relative abundance for a given ASV is shown in red.

Supplementary Figure 2 Unrooted phylogenetic relationship among ASVs not classified to Domain. A member of each Domain is included for reference: Archaea (*Halobacterium salinarum*), Eukaryota (*Collimonas fungivorans*) and Bacteria (*Basidiobolus heterosporus*).

Supplementary Figure 3 Phylogenetic tree of 16S rRNA gene sequences representing novel genera within the phylum Armatimonadota, rooted to *Chloroflexus aurantiacus*. Numbers 1 through 37 represent phylogenetic positions of proposed novel genera. The classified genera *Armatimonas*, *Capsulimonas*, *Fimbriimonas*, and *Chthonomonas* are included as reference.

Supplementary Figure 4 Phylogenetic tree of 16S rRNA gene sequences from classified sequences and novel genera in the phylum Pseudomonadota, rooted to *Chloroflexus aurantiacus*. Numbers 1 through 46 represent phylogenetic positions of proposed novel genera. A sample of classified genera are included for reference.

Supplementary Figure 5 Phylogenetic tree of 16S rRNA gene sequences from classified sequences and novel genera in the phylum Verrucomicrobiota, rooted to *Chloroflexus aurantiacus*. Numbers 1 through 58 represent phylogenetic positions of proposed novel genera. A sample of classified genera are included for reference.
