# Supplementary material for: Searching for bacterial plastitrophs in modified Winogradsky columns
Source: Front Microbiomes. 2024 Mar 20;3:1303112. doi: 10.3389/frmbi.2024.1303112 (PMC12993582; doi:10.3389/frmbi.2024.1303112)
Supplement: Supplementary Figure 1 — Individual ASVs that are significantly different in their relative abundances in plastic strips versus landfill soil. Five ASVs (A, B, E-G) showed significantly greater abundance in plastic-strip samples, and two (C, D) were significantly more abundant in landfill-soil samples. Relative abundance refers to the proportion of sequencing reads assigned to either plastic strips or landfill soil for each ASV, averaged over all plastic-strip (16) or landfill-soil (16) communities sampled in the columns. Differences in relative abundance were tested by the Wilcoxon Rank test and FDR adjusted p-value< 0.05. The substantially greater representation of five of the ASVs on plastic strips suggests they may have plastic-degrading capabilities. The ASVs indicated here with greater relative abundance on plastic strips represent novel genera within the Verrucomicrobiota (A, B) and the Pseudomonadota (G), or a novel phylum (E, F) Taxonomic classification and differential abundance analysis expand the known diversity of microbes associated with polyethylene degradation. Asterisks (*) represents the outliers for each ASV. The community type with greater relative abundance for a given ASV is shown in red. [file DataSheet_1.pdf]

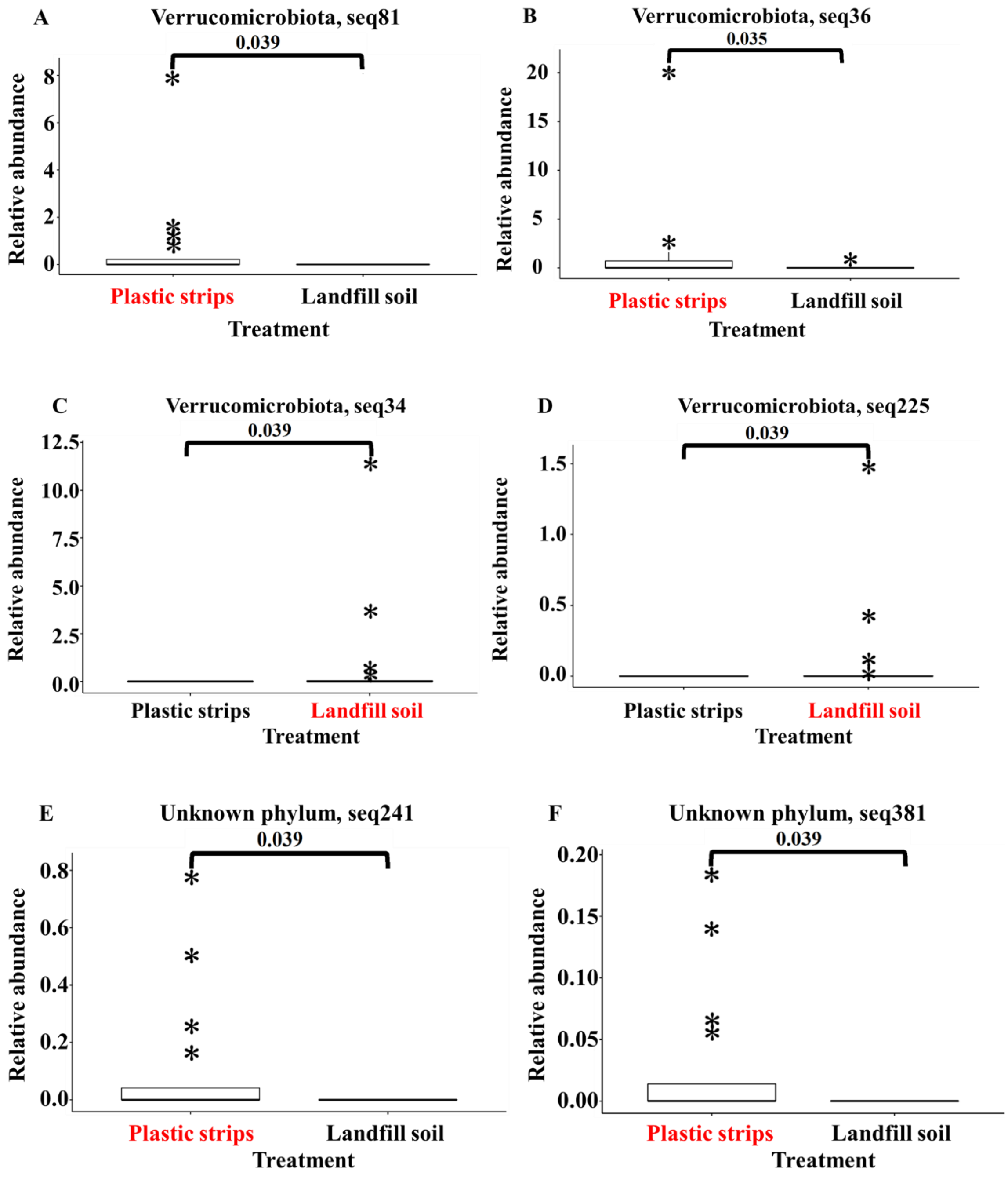

A
Verrucomicrobiota, seq81
0.039
Relative abundance
0
2
4
6
8
Plastic strips
Landfill soil
Treatment
B
Verrucomicrobiota, seq36
0.035
Relative abundance
0
5
10
15
20
Plastic strips
Landfill soil
Treatment
C
Verrucomicrobiota, seq34
0.039
Relative abundance
0.0
2.5
5.0
7.5
10.0
12.5
Plastic strips
Landfill soil
Treatment
D
Verrucomicrobiota, seq225
0.039
Relative abundance
0.0
0.5
1.0
1.5
Plastic strips
Landfill soil
Treatment
E
Unknown phylum, seq241
0.039
Relative abundance
0.0
0.2
0.4
0.6
0.8
Plastic strips
Landfill soil
Treatment
F
Unknown phylum, seq381
0.039
Relative abundance
0.00
0.05
0.10
0.15
0.20
Plastic strips
Landfill soil
Treatment

G

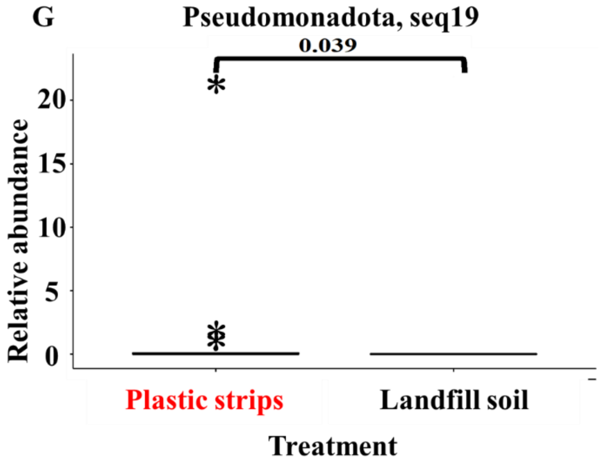

Supplementary Figure S1.

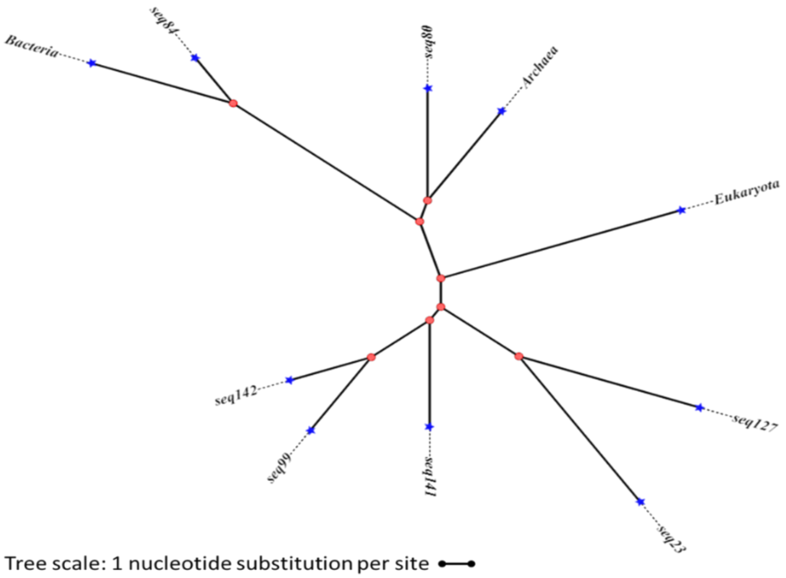

Supplementary Figure S2.

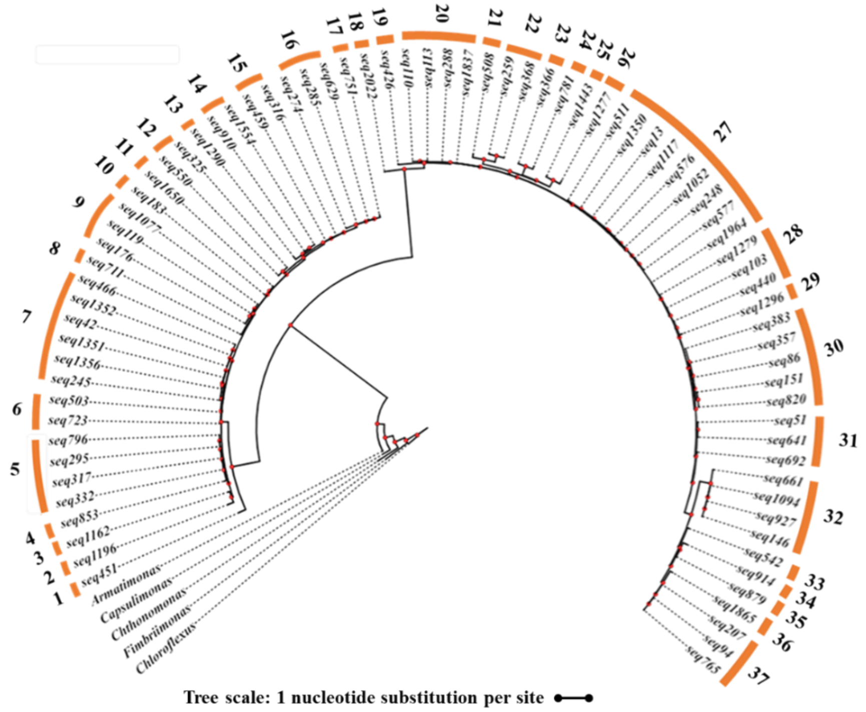

Supplementary Figure S3.

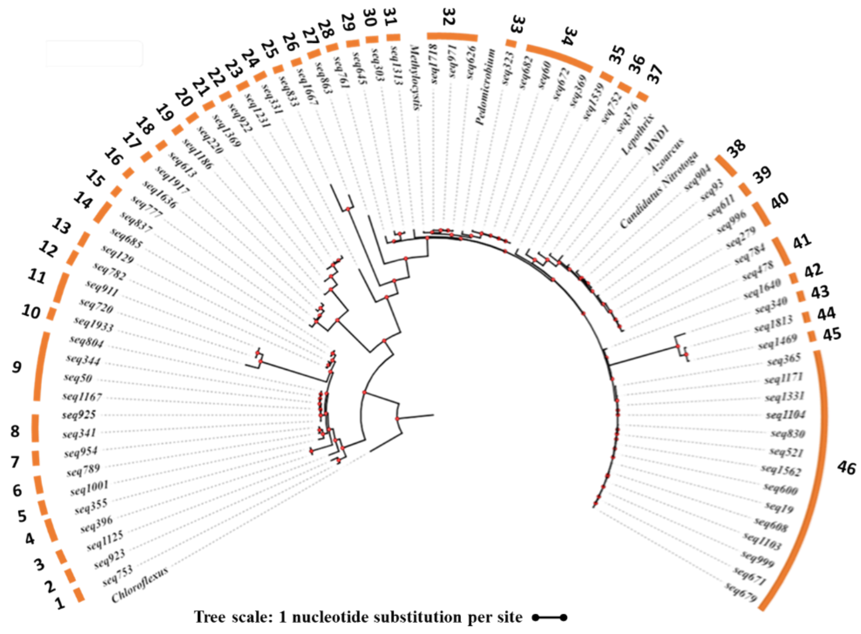

Supplementary Figure S4.

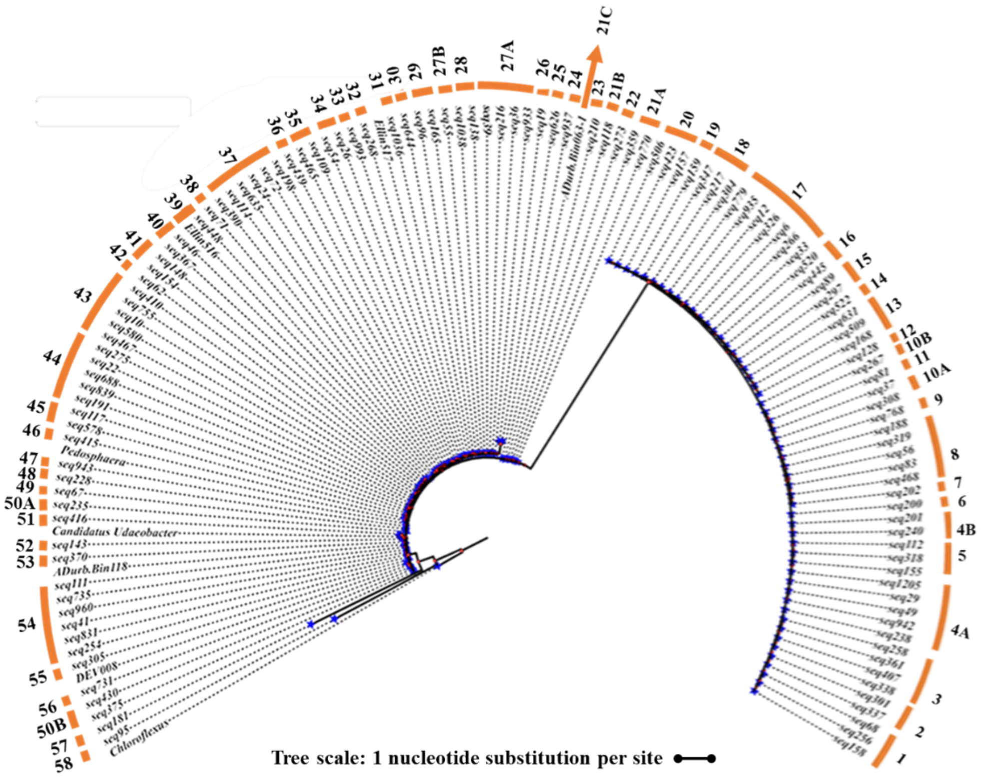

Supplementary Figure S5.

Supplementary Table S1. Full names, KO number, Log(Ratio), and P-value of proteins listed in Figure 4.

Description	Function	Log(Ratio)	P-value
**Two-component system, cell cycle sensor histidine kinase (pleC)**	K07716	0.34073705	0.033291
Carboxymethylenebutenolidase	K01061	0.33506751	0.010341
Amidase (amiE)	K01426	0.31753754	0.054283
Uncharacterized protein	K07080	0.30103722	0.015175
Uncharacterized protein	K07006	0.29494227	0.009477
Acyl-ACP dehydrogenase (mbtN, fadE14)	K00257	0.2877706	0.028427
MFS transporter, DHA2 family, multidrug resistance protein (emrB)	K03446	0.23427016	0.038448
2-(1,2-epoxy-1,2-dihydrophenyl)acetyl-CoA isomerase (paaG)	K15866	0.22961197	0.046158
3-hydroxybutyryl-CoA dehydrogenase (paaH, hbd, fadB, mmgB)	K00074	0.21847663	0.049683
N-substituted formamide deformylase (nfdA)	K07047	0.20973148	0.006581
MFS transporter, DHA1 family, multidrug resistance protein (bcr, tcaB)	K07552	0.20711201	0.039563
Cytochrome c-type biogenesis protein (ccmH)	K02200	0.19507045	0.004688
[Ribosomal protein S5]-alanine N-acetyltransferase (rimJ)	K03790	0.17976604	0.025998
Uncharacterized protein	K06978	0.1734366	0.013215
4-hydroxy-2-oxoheptanedioate aldolase (hpal, hpcH)	K02510	0.17309169	0.032742
Putative endonuclease	K07461	0.17269136	0.027509
Aminobenzoyl-glutamate utilization protein B (abgB)	K12941	0.17084299	0.022155
Butyryl-CoA dehydrogenase (ACADS, bcd)	K00248	0.16325912	0.033522
Lrp/AsnC family transcriptional regulator (ybaO)	K05800	0.16264631	0.048906
Citrate lyase subunit beta / citryl-CoA lyase (citE)	K01644	0.16229791	0.046158
